# Influential Women in the Field of Neurological Rehabilitation: A Literature Review

**DOI:** 10.3390/ijerph19031112

**Published:** 2022-01-20

**Authors:** Roberto Cano-de-la-Cuerda

**Affiliations:** Department of Physical Therapy, Occupational Therapy, Rehabilitation and Physical Medicine, Faculty of Health Sciences, Universidad Rey Juan Carlos, 28922 Madrid, Spain; roberto.cano@urjc.es; Tel.: +34-914888674

**Keywords:** authors, female, neurorehabilitation, neurological rehabilitation, researchers, women

## Abstract

Background: Medicine requires the brightest minds, regardless of gender. Women working in the health sciences have time and again demonstrated the value of their technical training, communication skills, emotional support, and ability to provide understandable explanations to their patients. The objective of this work was to carry out a historical review of the main female authors linked to classic sensorimotor neurorehabilitation techniques throughout the nineteenth and twentieth centuries, as well as female authors linked to eponymous tests or assessments, exposing their scientific trajectory and main contributions to the field of neurological rehabilitation. A literature review was conducted. The databases of Physiotherapy Evidence Database (PEDro), Scopus, CINAHL Medical Science, Medline through EBSCO and PubMed were used to obtain the biographical information of each author, searches of papers were limited until August 2021 in English and Spanish languages. Seventeen female authors were identified who linked to the main rehabilitation techniques or approaches described for neurological rehabilitation and for scales or tests with an eponymous origin as an example of female contribution on neurorehabilitation. Biographical information based on the computerized search in the electronic databases showed 57 potentially relevant articles. Of those articles, 43 were subsequently excluded. Fourteen articles were used to show their contribution to neurorehabilitation. This paper demonstrates the influential role of women in the history of sensorimotor neurorehabilitation throughout the nineteenth and twentieth centuries, linked to the methods, techniques, concepts, or approaches used in physical therapy or occupational therapy.

## 1. Introduction

Medicine requires the brightest minds, regardless of gender. The incorporation of women into the health sciences has helped to humanize them, thanks to their unquestionable technical training, their communication skills, emotional support, and their ability to provide understandable explanations to their patients [1]. However, women have difficulty in accessing leadership positions both in the clinical field and in academic and research areas since they do not advance as quickly, and are not rewarded in the same way, as their male colleagues who have undergone the same training [2]. Many relate this to the role that women have played culturally; the archaic belief that women do not have the physical or mental capacity to withstand certain workloads and the stress that such workloads impose; the fear of maternity leave; or the notion that women are not good negotiators and are less likely to seek positions of power due to insecurities or personal situations [3,4].

Gradually, medical specialties have ceased to differentiate, although some remain known colloquially as “feminized” (for example dermatology, ophthalmology, genetics or family medicine) and others as “masculinized” (for example general surgery, orthopedics, emergency medicine and neurosurgery). This phenomenon has been described as “the illusion of choice” with regard to medical specialties, where it is precisely gender bias that exists in their choice [5]. Despite nowadays being more inclusive, medicine has moved from integration to discrimination and there has been talk of a “silent bias” that applies to women in medicine seeking academic or high-command positions [5].

The lack of opportunities for women in leadership positions works like a “leaky pipeline” because despite having the knowledge and preparation, they simply get lost in the process and do not reach the top. This phenomenon is also often linked to the difference in the number of publications authored by women doctors who dedicate most of their time to the clinic rather than to research, though the lack of opportunities that academics have to publish compared to their peers has also been described [5,6]. However, even in those medical specialties considered “feminized”, and for women in the health sciences who have bequeathed eponymous (a name that is used to refer to a people, concept or object of any kind) techniques, scales or tests, or devised and developed therapeutic concepts that are studied today in all Faculties and Schools related to neurorehabilitation, they are unconsciously linked to a male author. With minimal exceptions, history has not yet recognized the role women have played in the field of neurorehabilitation.

As such, the objective of this work was to carry out a historical review of the prominent female authors linked to classic sensorimotor neurorehabilitation techniques throughout the nineteenth and twentieth centuries, as well as female authors linked to test or assessments described as eponymous, exposing their scientific trajectory and main contributions to the field of neurological rehabilitation.

## 2. Materials and Methods

A literature review was conducted. For this paper, searches focused on female authors who were linked to classic sensorimotor neurorehabilitation techniques or approaches described in Table 1, based on a previous review by Florez [7] and scales, test or assessments described as eponymous outlined in Table 2, based on [8,9,10,11]. One primary researcher carried out said article screening, data extraction and author identification. 

Biographical information and a brief contribution to neurorehabilitation were described for each author by a computerized search of the following electronic databases: CINAHL Medical Science, Medline through EBSCO, PubMed, Physiotherapy Evidence Database (PEDro) and Scopus. The search was limited to papers published until August 2021 in English and Spanish. The following keywords were used: *proprioceptive neuromuscular facilitation; Bobath concept; Brunnstrom technique; Rood Method; Perfetti Method; Task-oriented motor learning; Body-weight–supported treadmill training; Constraint-induced movement therapy; Muscle strengthening programs;* and *physical reconditioning*. Information about psychological rehabilitation, pharmaceutical or other medical interventions was excluded.

## 3. Results

Seventeen female authors were identified who were linked to the main rehabilitation techniques or approaches described for neurological rehabilitation and for scales or tests with an eponymous origin, based on Table 1 and Table 2. Biographical information based on the computerized search in the electronic databases showed 57 potentially relevant articles. Of those articles, 43 were subsequently excluded. Fourteen articles fulfilled all criteria for selection and were used to demonstrate their contribution to neurorehabilitation.

The order of the authors is not subject to any implicit meaning:

Florence Peterson Kendall was a physical therapist considered the “mother” of physical therapy. Florence spent many years treating polio patients at Children’s Hospital in Baltimore. In the 1940s, she was a supervisor of physical therapy for the Maryland State Department of Health, specializing in polio patients. Her book “*Muscles, Testing and Function*” has become one of the main textbooks for physical therapy students. Florence came to link muscle function with posture and pain. Drawing primarily on her extensive work researching people with polio, Florence’s book set a new standard for musculoskeletal assessment and treatment [12]. Kendall’s 0–10-point scale for manual muscle testing remains widely known.

Lucille Daniels and Catherine Worthingham were two physiotherapists who co-authored two of the most important books in rehabilitation: Muscle Testing (1946) and Therapeutic Exercise (1957). Daniels was an associate professor and director of the Division of Physical Therapy at Stanford University School of Medicine. Worthingham worked in the field of corrective physical education. In 1939, she was an assistant professor and director of physical therapy at Stanford University. She would later become a director at the National Foundation for Infantile Paralysis. In 1950, Worthingham was the first physical therapist to hold a doctoral degree (in anatomy). These two women systematized and defined the most commonly accepted method of evaluating muscle strength against the rater´s resistance using a 0 to 5 scale [13,14,15]. Colloquially, this testing is known as the Daniels and Worthingham Test and is another eponymous example within the history of health sciences.

Anne G. Fisher is an internationally recognized expert in occupational therapy. She was a pioneer in the application of the Rasch analysis in occupational therapy, and she developed the Occupational Therapy Intervention Process Model [16]. One of her assessments designed and commonly used with people with neurological disorders is the Assessment of Motor and Process Skills (AMPS). This is an observational tool for the assessment of an individual’s ability to perform complex or instrumental and personal activities of daily living [17].

Cynthia C. Norkin was a physical therapist, an associate professor emerita and director of the School of Physical Therapy at the College of Health and Human Services, Ohio University, Athens, Ohio. She held a doctoral degree (in education) from Boston University in 1984. She authored the most important book about the measurement of joint motion in rehabilitation, which is commonly used in people with neurological disorders. She systematized the assessment of range of motion using universal goniometers and inclinometers [18].

Mary E. Tinetti is the Gladys Phillips Crofoot Professor of Medicine and Public Health, as well as Chief of Geriatrics at Yale School of Medicine [19]. Through her research, she determined that older adults at risk of falling could be identified with her internationally known developed scale, the eponymous Tinetti Performance Oriented Mobility Assessment [20]. This assessment is one of the most prominently used in evaluating postural control in people with neurological disorders such as multiple sclerosis, Parkinson´s disease, or stroke [21].

Katherine Berg is a Canadian physical therapist. She holds a doctoral degree, the thesis of which was titled “*Measuring balance in the elderly: development and validation of an instrument*”. Her research interests include disability and fall prevention as well as health services studies examining quality of care and outcomes. She is eponymously associated with the Berg Balance Scale [22], used to measure balance in the elderly. It is widely used in people with neurological disorders and has been cross-culturally translated into different languages. She is chair and associate professor in the Department of Physical Therapy and the Graduate Department of Rehabilitation Science, Faculty of Medicine at University of Toronto, as well as a fellow with interRAI [23,24].

Jacquelin Perry was an orthopedic surgeon and leading authority on gait analysis. She was born in Denver, Colorado, USA on 31 May 1918, and died in Downey, California on 11 March 2013. When Perry found herself obliged to stop operating for health reasons, she followed the advice she was accustomed to giving to her patients: accommodate your new reality. With a prior qualification in physical therapy and already interested in human movement, her own accommodation of reality was to set up a gait laboratory at Rancho Los Amigos National Rehabilitation Center in California. Perry first established a national and then an international reputation as one of the leading authorities on the biomechanics of walking and other forms of movement. During the 1980s, Perry was among the first to closely study what became known as post-polio syndrome [25]. In 1992, she wrote “*Gait Analysis: Normal and Pathological Function*”, the classic text on gait analysis. Dr. Perry was a visionary pioneer in the field of rehabilitation sciences. She was honored with “Woman of the Year for Medicine” by the Los Angeles Times in 1959 [26].

Margaret S. Rood, applied neurophysiological research findings to children and adults with motor impairments. The Rood method proposes achieving greater control of voluntary movement and posture based on the physiological differences of motor units. According to Rood, selecting the sensory inputs that reach the body can influence the structures of the CNS and improve muscle activity for greater control of movement and posture. In line with this physiological basis, different types of muscle fiber can be stimulated depending on the intensity of the applied stimulus. The Rood method includes different techniques to facilitate movement and normalize muscle tone, as follows: facilitating techniques (quick and gentle brushing, brief application of ice, sensory stimulation, rapid tapping, sustained pressure, vestibular stimulation); and normalizing techniques (prolonged application of ice, vibrations, slow stretching, slow passive mobilizations, percussion on the antagonist tendon, sustained pressure, vestibular stimulation) [11]. Rood was awarded the Eleanor Clarke Slagle Lectureship in 1958 [27].

Anna Signe Sofia Brunnstrom was born in Stockholm, Sweden, on 1 January 1898. She died in Darien Convalescent Center in Darien, Connecticut on 21 February 1988 [28]. The method proposed by Brunnstrom, known as the Brunnstrom Method, is one of the classic techniques of neurological physiotherapy, though at present it is rarely applied due to the controversy it has generated. This technique proposed the stimulation of synergistic movement patterns through the use of reflexes, associated reactions, and afferent stimuli, without voluntary effort. Later, these synergies would be modified to allow the approach to a more normal movement, owing to the decrease in muscle tone and increased voluntary control. Brunnstrom described seven recovery phases of the hemiplegic patient, during which the patient would theoretically gradually acquire greater voluntary control of movement, from the flaccid phase that immediately follows the stroke to the last phase, in which the patient is already able to perform all types of independent movements with the upper extremities, as well as selective movements of the lower extremities [29,30].

Berta Bobath was a German physiotherapist. She was born in Berlin on 5 December 1907. Initially, she studied at the “Anna Hermann” gymnastics and dance school and, after completing her training, she remained at this school as an instructor until 1933. In 1938, she went to Germany and following that, took up residence in London. In London she began to work as a gymnastics instructor. In 1941, she gained employment at The Princess Louise Hospital for Children and studied to become a physiotherapist. She married the physician Karel Bobath in 1941 and, from the neuroscience available at the time, together they developed the Bobath Concept. This is an approach to the assessment and treatment of individuals with impaired function, movement, and postural control due to a central nervous system injury. The Bobath Concept is based on the modulation of abnormal reflex activities and the facilitation of normal movement patterns through key control points, always directed to a specific functional objective. The treatment is globalizing, and part of an approach whose first step is the analysis of the norm. They both taught and provided training in this concept for different rehabilitation professionals throughout the world. It is the most popular approach for treating patients with neurological disorders in the Western world. Berta Bobath passed away on 20 January 1991 [30].

Margaret Johnstone was born in 1919, eventually becoming a physiotherapist and Founder of the Johnstone Approach. Johnstone graduated from the Royal Infirmary in Edinburgh in 1943. She proposed learning as a lifelong process, reflected in her work of searching for methods of recovery for stroke patients in their rehabilitation process. Initially, she accumulated experience with orthopedic patients, children and polio patients. In 1966, Margaret started to use inflatable air splints as a simple way to treat upper limbs in people with stroke. Margaret paid specific attention to keeping the hemiplegic limb out of compensatory patterns and the detrimental movements of the low-motor stroke patient to a minimum [11]. This could be regarded as an early idea of “forced use“, and certainly as pioneering work, now developed further by theories of contemporary motor control and motor learning. Currently, these splints are used in brain damage units worldwide under her motto: “the air splints are only tools. The result of an effective rehabilitation program depends on how you use them”. She wrote five books which were also translated in various languages. She died on 13 April 2006 in Peeblesshire, Scotland [11].

Jean Ayres was an occupational therapist who developed a theory of sensory integration, which she believed to be basic to children’s ability to be successful in everyday activities, and was an advocate for individuals with special needs. She developed the psychometric properties of tests of various aspects of sensory integration, and invented equipment to be used in practice. Ayres inspired occupational therapists to become researchers both by role modeling and by teaching graduate students. She authored several books that are used worldwide [31].

Margaret Knott was a physical therapist who became known worldwide for teaching the proprioceptive neuromuscular facilitation (PNF) technique, first described for polio, multiple sclerosis and rheumatic fever patients. Dr. Kabat, Director of the Kabat-Kaiser Institute (Vallejo, CA, USA), together with physiotherapist Margaret Knott and later with Dorothy Voss in 1953, expanded and developed the PNF method nationally and internationally. In 1956, Knott and Voss published the first textbook on PNF. Margaret Knott passed away in 1978. The philosophy and basic principles of PNF, together with the specific spiral and diagonal patterns, are the essential ideas linked to PNF. All activities within PNF intervention are directed towards a functional objective and are relative to the environment in which the function is to be achieved [32,33]. PNF has become internationally recognized and widely used as an effective treatment for certain injuries and illnesses, many of them of the CNS. 

Janet Howard Carr and Roberta Barkworth Shepherd are two Australian physiotherapists who, in 1984, based on studies of movement science, neurophysiology and learning theories, proposed a new approach to the rehabilitation of stroke patients. According to Carr and Shepherd, the goal of rehabilitative treatment should be relearning oriented to specific tasks, forcing the use of the paretic side. Their principle was simple and intuitive: “one learns what one practices” [34,35]. Activity-oriented methods rely on the recognition that the goal of motor control is mastery of movement to perform a particular action, not to perform movements for the sake of moving. Thus, movement control would be organized around goal-directed functional behaviors. Other modern techniques such as bodyweight-supported treadmill training and constraint-induced movement therapy, or technologic approaches such as robotics, virtual reality, and video games, are based in this clear aphorism proposed by Carr and Shepherd.

Finally, Clare S. Spackman was a pioneer in occupational therapy. She was the author and co-editor, alongside Helen Willard, of the first book in occupational therapy, “*Principles of Occupational Therapy*”, published in 1947. She served as secretary from 1952 to 1956 and president from 1957 to 1962 of the World Federation of Occupational Therapists. She was an active clinician at the Curative Workshop of Philadelphia and an academic educator at the University of Pennsylvania School of Allied Health Professions [36].

## 4. Discussion

The role of women in medicine has progressively increased over time, starting from a highly competitive and predominantly male context, and moving to the distinguished collegiate physicians of present day. The notable interest of women in medical careers was promoted at the end of the nineteenth century, within the framework of a democratization process, such as the fight for the right to female suffrage, equal working conditions, and the right to education, among other social conquests [37,38].

The origins of neurological rehabilitation are potentially linked to polio epidemics. Elizabeth Kenny (1886–1952), a proponent of early mobilization, used humerus heat and therapeutic exercise in patients with poliomyelitis, whose treatment allowed the development of physiotherapy in the twentieth century, not only from movement therapy, but also through physical agents such as electricity, light, and heat [30]. Furthermore, many of the therapeutic concepts that went on to be developed at that time were based on Elisabeth Kenny’s treatment premises of the early mobilization of patients and the importance of active participation.

Contemporary with Elizabeth Kenny was the British woman Olive Frances Guthrie Smith (1883–1956), a physiotherapist at St Mary’s Hospital in London, previously trained during the First World War in the “Almeric Paget (Military) Massage Corps,” an institution that would treat the injuries of wounded soldiers. Olive Guthrie Smith was practically the inventor of suspension therapy, used in the treatment of neurological patients in the first half of the twentieth century, which lost its place in rehabilitation services over time [30].

In the 1950s, a major conceptual shift in neurological physical therapy was evident as the neurophysiological or neurofacilitation approaches were developed, mainly linked to female researchers and therapists. The focus changed from muscle to non-muscle elements. Techniques were primarily directed at the nervous system, with movement facilitation by stimulation of the nervous system. Major influences were the work of the Bobath Concept and that of Kabat, Knott, and Voss (PNF). Other therapists also developed their ideas for therapy around this time, including Rood, Ayres, and Brunnstrom. These approaches are often referred to as eponymous as they were named after their originators [39], all of them related to female researchers.

Neurological rehabilitation has changed over the past decades as scientific and technological developments have offered a greater understanding of brain reorganization and the mechanisms of motor control, performance, impairments, and adaptations, owing to the many female researchers described in this paper. Current scientific research is leading to a changing focus in clinical interventions, with emphasis on optimizing motor performance through task-oriented exercise and training, strength training, and taking the influence of the environment into account. In this context, to achieve successful rehabilitation, increased emphasis needs to be placed on challenging, motivated, and meaningful task training to promote learning, as many of the female authors such as Carr and Shepherd highlighted, and as described in the Results section of the present paper [39].

The influential role of women in the history of neurorehabilitation is unquestionable, as most of the classic methods, techniques, concepts, or approaches used today in neurorehabilitation (Table 1) were described and/or developed by female researchers linked to physical therapy or occupational therapy. Finally, since neuroscience findings can have major implications for treatment approaches, the rapid incorporation of these ideas into neurorehabilitation is essential. The facilitation of this translation by therapists who are well-versed in these ideas will aid the development of the profession and facilitate improvements in patient care [40].

To achieve these objectives, it is important to know the historical contributions of the pioneers and contemporary prominent authors on neurorehabilitation, and to promote gender equity at all levels including clinical, teaching, research, and management, since an underrepresented situation at multiple levels for females in key areas of neurorehabilitation has been documented [41,42,43,44,45].

This historical review presents several limitations. First, many other female researchers have significantly contributed to the development of neurorehabilitation to its present stage. It has been presented an approach regarding the primary contributions from the developmental to contemporary period to neurological rehabilitation. Conversely, no aspect related to psychological rehabilitation has been addressed in this review. Finally, the language restriction of the literature reviewed must be noted as other important authors could be missing in this review.

## 5. Conclusions

The role of women in medicine has been progressively increasing over time, starting from a highly competitive and predominantly male context, and moving to the distinguished collegiate physicians of present day. A historical review of the prominent women authors linked to sensorimotor neurorehabilitation throughout the nineteenth and twentieth centuries was presented, exposing their scientific trajectory and main contributions to the field of neurological rehabilitation. In this context, the influential role of women in the history of neurorehabilitation is undeniable, given that many of the classic methods, techniques, concepts, and approaches used in neurorehabilitation were described and/or developed by female researchers linked to physical therapy and occupational therapy. 

## Figures and Tables

**Table 1:** Historical and conceptual classification of techniques in neurorehabilitation.

	Description	Aim	Approaches and Techniques
<1940	Compensation techniques	Stimulate unaffected side	
1940–1960	Facilitation techniques	Improve quality of movement on the affected side	Proprioceptive neuromuscular facilitation 1940 Bobath concept 1948 Brunnstrom 1950 Rood Method 1954
>1970	Neurocognitive techniques	Introduction of cognitive functions in the rehabilitation process	Perfetti Method 1970
>1970–1980	Modern techniques	Motor learning	Task-oriented motor learning 1984 Body-weight–supported treadmill training 1987 Constraint-induced movement therapy 1970–1980 Muscle strengthening programs and physical reconditioning 1970–1980

Modified from [7].

**Table 2:** Main scales, tests and assessments in neurorehabilitation.

Scales related to alterations in structure or function	Beck Depression Inventory Behavioral Inattention Test Canadian Neurological Scale Clock Drawing Test Frenchay Aphasia Screening Test Fugl-Meyer Assessment of Motor Recovery after Stroke General Health Questionnaire–28 Geriatric Depression Scale Hospital Anxiety and Depression Scale Mini-Mental State Examination Modified Ashworth Scale Montreal Cognitive Assessment Motor-free Visual Perception Test National Institutes of Health Stroke Scale Orpington Prognostic Scale Penn Scale Tardieu Scale
Scales related to alterations in activity	Ten Meter Walk Test Six-Minute Walk Test Action Research Arm Test Barthel Index Berg Balance Scale Box and Block Test Chedoke–McMaster Stroke Assessment Scale Clinical Outcome Variables Frenchay Inde Functional Ambulation Categories Functional Independence Measure Functional Reach Test National Rehabilitation Reporting System Frenchay Activities Index (FAI) Get Up and Go Test Timed Get Up and Go Test Grooved Pegboard Fugl Meyer Assessment Hauser Inde Jebsen Taylor Test of Hand Function Lawton and Brody Scale Modified Rankin Handicap Scale Motor Activity Log Motor Assessment Scale Motricity Inde Nine-hole Peg Test Purdue Pegboard Test Rivermead Mobility Index Timed “Up & Go” Test Tinetti Test Trunk Control Test Wolf Motor Function Test
Scales related to participation restrictions	Canadian Occupational Performance Measure EuroQol Quality of Life Scale London Handicap Scale Medical Outcomes Study Short Form 36 Nottingham Health Profile Reintegration to Normal Living Index Stroke-Adapted Sickness Impact Profile Stroke Impact Scale Stroke Specific Quality of Life Scale

Modified from [8,9,10,11].
